# *Dendrobium officinale* Endophytes May Colonize the Intestinal Tract and Regulate Gut Microbiota in Mice

**DOI:** 10.1155/2022/2607506

**Published:** 2022-08-11

**Authors:** Wenhua Chen, Lilong Yu, Bo Zhu, Luping Qin

**Affiliations:** School of Pharmaceutical Sciences, Zhejiang Chinese Medical University, Hangzhou 310053, China

## Abstract

*Dendrobium officinale* is a traditional Chinese medicine for treating gastrointestinal diseases by nourishing “Yin” and thickening the stomach lining. To study whether *D. officinale* endophytes can colonize the intestinal tract and regulate gut microbiota in mice, we used autoclave steam sterilizing and ^60^Co-*γ* radiation to eliminate *D. officinale* endophytes from its juice. Then, high-throughput ITS1-ITS2 rDNA and 16S rRNA gene amplicons were sequenced to analyze the microbial community of *D. officinale* endophytes and fecal samples of mice after administration of fresh *D. officinale* juice. Sterilization of *D. officinale* juice by autoclaving for 40 min (ASDO40) could more effectively eliminate the *D. officinale* endophytes and decrease their interference on the gut microbiota. *D. officinale* juice could increase beneficial gut microbiota and metabolites including short-chain fatty acids. *D. officinale* endophytes *Pseudomonas mosselii*, *Trichocladium asperum*, *Titata maxilliformi*s, *Clonostachys epichloe*, and *Rhodotorula babjevae* could colonize the intestinal tract of mice and modulate gut microbiota after oral administration of the juice for 28 days. Thus, the regulatory effect of *D. officinale* juice on gut microbiota was observed, which provides a basis for inferring that *D. officinale* endophytes might colonize the intestinal tract and participate in regulating gut microbiota to treat diseases. Thus, this study further provides a new approach for the treatment of diseases by colonizing plant endophytes in the intestinal tract and regulating gut microbiota.

## 1. Introduction

*Dendrobium officinale* Kimura et Migo (*D. officinale*), recorded in Chinese Pharmacopoeia, is used as herbal medicine and novel food material in China [1, 2]. It is widely used as a traditional medicine to strengthen “Yin” (“Yin” is one of the two complementary opposite forces of nature, per the ancient Chinese construct of the universe. “Yin” is characterized as slow, soft, yielding, diffuse, cold, wet, or tranquil, and it is associated with water, earth, the moon, femininity, and nighttime.), which can tonify the five viscera, relieve fatigue, thicken stomach lining, lighten the body, and prolong life span [3]. According to modern pharmacological studies, *D. officinale* exhibits various biological functions, including balancing gut microbiota [4], immune modulation [5], antitumor [6], gastrointestinal protective [7], cardioprotective [8], and antidiabetes effects [9]. Based on currently available phytochemical investigations, polysaccharides, bibenzyls, phenanthrenes, flavonoids, and alkaloids are the major bioactive constituents of *D. officinale* [3]. Several recent studies have shown the role of gut microbiota in mediating the health and disease of the host [10]. Hence, balancing the effect of gut microbiota should be paid more attention to in the prevention and treatment of diseases [4].

The gut microbiota consists of trillions of bacteria and fungi and is profoundly important in maintaining human health because of its role in nutrient acquisition and energy regulation [11]. Microbes that colonize inner plant tissues are designated endophytes. Endophytes exist widely in host plants and are important components of plant microecosystems [12]. Endophytes have increasingly become the research hotspot of scholars worldwide because they can produce active components, promote host plant growth, and enhance the host plant resistance against biotic and abiotic stresses [13, 14]. Recent research has unearthed a network of endophytic-enteric-soil-endophytic microbes that process animal feces to serve as natural microbial inoculants for plants. These function to serve as bacterial sources for animal gut systems [15]. Gut microbiota contains hundreds to thousands of bacteria obtained from a specific diet [16]. Li et al. (2012) found that *Paenibacillus* sp. strain Aloe-11 had excellent intestine colonization ability and could significantly promote forage fiber degradation, thus producing antibiotic activity against many pathogenic bacteria and fungi [17]. Likewise, Zheng et al. (2020) found that a proportion of the intestinal microbes of potato tuber moth might be derived from bacterial endophytes in potatoes [18]. In another report, it was shown that fungal endophytes in grass eaten by sheep could reach the gut and reduces fecal degradation rates [19].

*D. officinale* can act as a prebiotic agent to promote short-chain fatty acids (SCFAs)-producing genera and avoid gut dysbiosis in dogs [11]. *D. officinale* can also inhibit the growth of pathogenic bacteria by increasing the SCFAs-producing beneficial bacteria, showing anti-inflammatory activity, and improving the human intestinal environment [20]. While potato endophytic bacteria can colonize and transform into intestinal microbes in potato tuber moths [18], little is known about whether *D. officinale* endophytes can colonize and transform the intestinal tract and play a similar role in regulating gut microbiota in mice. In this study, we investigated the endophytic microbes (fungi and bacteria) of *D. officinale* that can colonize the intestinal tract and regulate gut microbiota in mice. This work will provide an important basis for studying the colonization of *D. officinale* endophyte in the intestinal tract of mice.

## 2. Materials and Methods

### 2.1. Material

*D. officinale* plant material was artificially cultivated in the base of Lin'an (30°23′ N, 119°72′ E), and was identified as *D. officinale* Kimura and Migo by Professor Qiaoyan Zhang of the Zhejiang Chinese Medicine University. The collected samples of *D. officinale* were packed in sterile plastic bags and brought back to the laboratory.

The collected samples of fresh *D. officinale* stems (DO) were cleaned under running water, surface-sterilized with 75% ethanol for 3 min, 5% NaClO for 3 min, 75% ethanol for 30 s, and finally washed five times with sterile distilled water [21]. Fresh DOs were treated with doses of 5, 10, 15, 20, and 25 kGy ^60^Co-*γ* irradiation (CIDO5, CIDO10, CIDO15, CIDO20, and CIDO25, respectively). Fresh DOs were treated in autoclaving steam sterilizers under set conditions and autoclaved at 121°C for 20 min (ASDO20) and 40 min (ASDO40). Three parallel copies of each sample were prepared to use 2 g of each sample and placed in a DNA-Be-Locked reagent (Majorbio Bio-Pharm, Shanghai, China) to immobilize DNA to study the endophytic bacteria and fungi.

Chemical reagents including acetic acid (99.7% purity), propionic acid (99.5% purity), butanoic acid (99.5% purity), isobutyric acid (99.0% purity), valeric acid (99.0% purity), isovaleric acid (99.0% purity), hexanoic acid (99.0%), and isohexanoic acid (99.0% purity) purchased from Sigma Corporation (St. Louis, MO, USA) were used as SCFAs standards. All other reagents were of analytical grade and purchased from Shanghai Chemicals and Reagents Co. (Shanghai, China).

### 2.2. Animal Experiments

All animal experiments were performed following the guidelines approved by the Committee on the Ethics of Animal Experiments of Zhejiang Chinese Medical University (SYXK—2018—0012). Eighteen 6-week-old C57BL/6 male mice were purchased from Shanghai Laboratory Animal Center (Shanghai, China). The animals were kept in three individual cages with free access to food and sterile drinking water in a temperature-controlled room (22 ± 2°C), relative humidity (50 ± 10%), and 12 h/12 h light/dark cycle.

After an adaption period of one week, the mice were randomly categorized into three groups, with six mice in each group. The mouse in DO and ASDO groups were administrated orally with fresh *D. officinale* juice and autoclaved fresh *D. officinale* juice (121°C for 40 min), respectively, at a dose of 1 g/kg daily for 28 days [22], whereas the control groups were orally administered sterile drinking water.

Fresh fecal samples of control (sterile water), DO, and ASDO (121°C for 40 min) groups were collected in sterile cryovial tubes for 0 (C0, DO0, ASDO0) and 28 days (C28, DO28, ASDO28). After 28 days, feces were stored at −80°C for the analysis of SCFAs and high-throughput sequencing.

### 2.3. Determination of SCFAs by Gas Chromatography-Mass Spectrometry (GC-MS)

The SCFAs were analyzed by GC-MS as previously described [23]. The analysis was carried out using an Agilent 8890B–5977 B system equipped with HP FFAP capillary column (30 m × 0.25 mm × 0.25 *μ*m; Agilent J&W Scientific, Folsom, CA, USA). The initial oven temperature was kept at 80°C for 0.5 min and then raised to 120°C at the rate of 40°C/min and 200°C at the rate of 10°C/min. The temperature of injection was 230°C for 3 min. Nitrogen was used as the carrier gas at a flow rate of 1.0 mL/min. The injected sample volume for GC analysis was 1 *μ*L. Electron bombardment ion source (EI) was used at a temperature of 230°C, four-stage rod temperature of 150°C, transmission line temperature of 230°C, and electron energy of 70 eV. The scanning mode was ion scanning mode (SIM). Calibration curves were constructed in the range of 0.2-400 *μ*g/mL (0.2, 1, 2, 10, 20, 100, 200, 400 *μ*g/mL) for acetic acid, propionic acid, and n-butyric acid, and 0.1-200 *μ*g/mL (0.1, 0.5, 1, 5, 10, 50, 100, 200 *μ*g/mL) for isobutyric acid, valeric acid, isovaleric acid, hexanoic acid, and isohexanoic acid (three replicates for each level), by adding known amounts of the analytes to the blank.

### 2.4. Genomic DNA Extraction, Polymerase Chain Reaction (PCR) Amplification, and Sequencing

Two plant samples (DO and ASDO40) and six fecal samples (C0, DO0, ASDO0, C28, DO28, and ASDO28) were selected to prepare the DNA of *D. officinale* endophytes and mice intestinal microorganisms [21]. In brief, total genomic DNA was extracted using the FastDNA 2 mL SPIN Kit for Soil (50 preps., Cat. No. 116560200, MP Biomedicals GmbH, Eschwege, Germany) and evaluated using a NanoDrop Spectrophotometer Qubit2.0.

The V5–V7 regions of the 16S rRNA genes of plants and bacteria in fecal samples were PCR amplified using universal primers for Illumina deep sequencing [18]. Primers 799 F (5′-AACMGGATTAGATACCCKG-3′) and 1193R (5′-ACGTCATCCCCACCTTCC-3′) outperform all other primer pairs in our study in the elimination of nontarget DNA and retrieval of bacterial OTUs [24]. In the plant and fecal samples, fungal internal transcribed spacer region 1 (ITS1 region) of ribosomal RNA was amplified using ITS1F (CTTGGTCATTTAGAGGAAGTAA) and ITS2R (GCTGCGTTCTTCATCGATGC) primers [12]. The PCR was carried out according to a protocol as described in our previous publication [21]. Library construction and sequencing were performed by Shanghai Majorbio Bio-pharm Technology Co., Ltd, (Shanghai, China).

### 2.5. Bioinformatic Analysis

The plant endophytic bacterial and fungal communities and fecal bacteria and fungi were subjected to the same analytical procedures. First, paired-end Illumina MiSeq sequences were merged using FLASH (v1.2.11, https://ccb.jhu.edu/software/FLASH/index.shtml) to obtain the raw tags [25]. These raw tags were then filtered and clustered using the Quantitative Insights into Microbial Ecology (QIIME) software v1.9.1 (http://qiime.org/install/index.html) [26]. Primers, short reads, low complexity reads, and low-quality sequences were removed using PRINSEQ v0.20.4 [27]. Sequencing and PCR-induced errors were corrected with the precluster function of the software Mothur v1.30.2 (https://www.mothur.org/wiki/Download-mothur) [28]. High qualified tags with ≥97% similarity were clustered into operational taxonomic units (OTUs) based on using software USEARCH v7 to the Greengenes v135 database for endophytic bacteria and fungi [29, 30]. Bacterial and fungal OTUs were classified by searching against the SILVA databases (Release 119, http://www.arb-silva.de), using the Ribosomal Database Project (RDP) classifier v2.11 (https://sourceforge.net/projects/rdp-classifier/) within QIIME [31, 32]. The analysis of variance (ANOVA), Multidimension, and Venn diagram with R software v3.3.1 based on OTUs was applied to compare the bacterial and fungal communities of each sample [33].

### 2.6. Statistical Analysis

All results were expressed as mean ± standard deviation (SD). Data were analyzed using one-way ANOVA followed by a Tukey's post hoc test using the SPSS 19.0 software (SPSS Inc., Chicago, IL, USA). *P* values less than 0.05 were considered of statistical significance.

A heatmap was employed to exhibit the relative abundances of the 50 predominant genera in each sample. The distance matrices of community composition of endophytic fungi and bacteria were evaluated by calculating dissimilarities using the Bray–Curtis method [34]. Beta diversity was estimated by calculating the weighted UniFrac distances between samples [35] using beta diversity by a program in QIIME. The data analysis for all correlations was finished in the online “i-sanger” (http://www.-majorbio.com/) developed by Majorbio Bio-Pharm Technology Co. Ltd [36].

## 3. Results

### 3.1. Autoclave Steam Sterilizing and ^60^Co-*γ* Treatment Eliminated the *D. officinale* Endophytes

**Figure 1:**
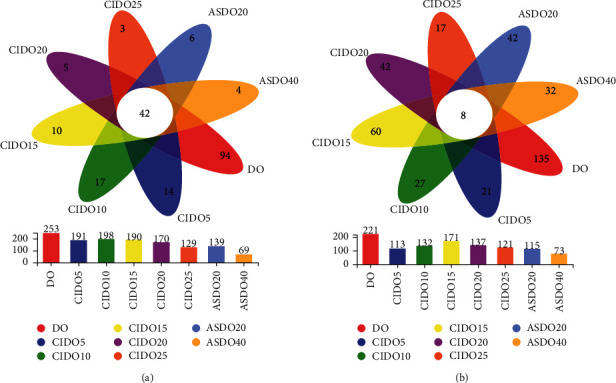
Venn diagram analysis for unique and shared operational taxonomic units among (a) bacteria and (b) fungi in *D. officinale* under different ^60^Co-*γ* irradiation and autoclave steam sterilizing treatments.

To eliminate the *D. officinale* endophytes and decrease their interference on the gut microbiota in mice, *D. officinale* was treated with autoclave steam sterilizing and 60Co-*γ*. After quality control, denoising, and removal of chimera sequences, 265,440 and 961,560 high-quality 16S and ITS1-ITS2 sequences were obtained. A total of 1,339 and 1,083 OTUs with 97% identity cutoffs of bacteria and fungi of *D. officinale* were found (Figure 1). The number of bacterial and fungal OTUs of *D. officinale* was reduced significantly after ^60^Co-*γ* irradiation and autoclave steam sterilizing treatment; autoclave steam sterilizing treatment (121°C for 40 min) decreased the OTUs most. Concurrently, the number of the phylum, class, order, family, genus, and species of bacteria (Supplement Figure 1(a)) and fungi (Supplement Figure 1(b)) of DO and ASDO40 decreased by 53.33%, 65.38%, 61.02%, 61.36%, 70.78%, and 64.97%, and 33.33%, 42.86%, 51.35%, 46.77%, 49.35%, and 44.68%, respectively. Overall, the sorts of *D. officinale* endophytic fungi and bacteria decreased the most significantly after 40 min autoclave steam sterilizing treatment.

**Figure 2:**
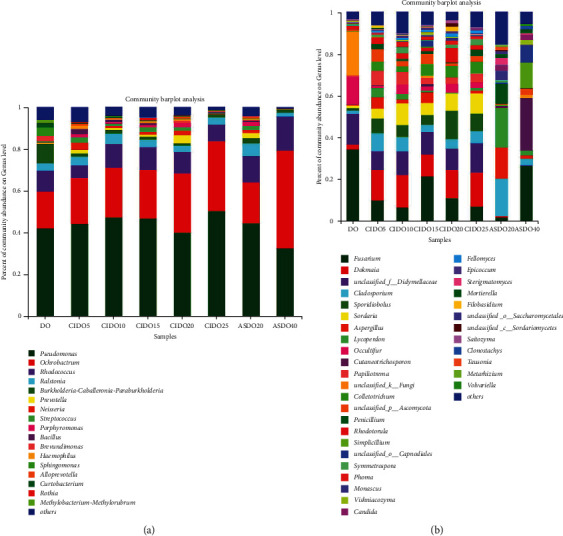
Relative abundance of the (a) bacterial and (b) fungal genus present in *D. officinale* with different ^60^Co-*γ* irradiation and autoclave steam sterilizing.

The larger the Chao, the higher the richness while the smaller the Shannon, the higher the diversity. Analysis of alpha diversity indicated that the community richness (Chao) and community diversity (Shannon) of endophytic bacteria and fungi in samples under autoclave steam sterilizing treatment (ASDO40) was lower than DO (Supplement Figures 2(a)–2(d)). However, ASDO40 treatment did not change the dominant bacterial genera compared to DO samples (Figure 2(a)). The top three dominant bacterial genera (*Pseudomonas*, *Ochrobactrum*, and *Rhodococcus*) in DO and ASDO40 samples were accounting for 69.45% and 95.48% of the relative abundance, respectively. However, in ASDO40, there was a decrease in the relative abundance of *Burkholderia*-*Caballeronia*-*Paraburkholderia*, *Alloprevotella*, *Prevotella*, *Neisseria*, and *Streptococcus*, compared with those in DO (Figure 2(a)). In addition, ASDO40 treatment changed the dominant fungal genera. The top three dominant fungal genera in DO were *Fusarium*, unclassified-f-Didymellaceae, and *Occultifur*, accounting for 62.68% of the relative abundance, while those in ASDO were *Fusarium*, *Cutaneotrichosporon*, and *Simplicillium*, accounting for 63.65% of the relative abundance. Furthermore, ASDO reduced the relative abundance of *Occultifur*, *Rhodotorula*, *Pyrenochaetopsis*, *Sporidiobolus*, and *Sordaria,* compared with those in DO (Figure 2(b)). Thus, ASDO40 treatment reduced the diversity and richness of *D. officinale* endophytes and participated in eliminating the interference of *D. officinale* endophytes.

### 3.2. Change in the Gut Microbiota Structure and Metabolite SCFAs after the Intake of *D. officinale* Juice

**Figure 3:**
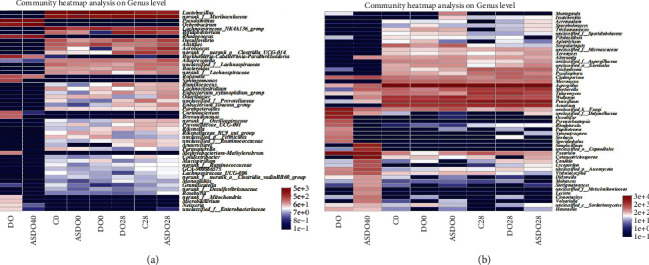
Community heatmap analysis of bacteria (a) and fungi (b) at the genus levels in fecal samples of mice.

To analyze how *D. officinale* juice modulates the gut microbiota structure in mice, we carried out 16S and ITS sequencing on fecal samples of mice administered with sterile water (C), *D. officinale* juice (DO), and autoclaved *D. officinale* juice (ASDO) on days 0 and 28. In all fecal samples, *Lactobacillus*, *Bifidobacterium*, and *Desulfovibrio* were the three top dominant bacterial genera, while *Aspergillus*, *Penicillium*, and *Acaulium* were the three top dominant fungal genera. Our experiments showed that oral administration of *D. officinale* juice for 28 days (DO28) could effectively increase the diversity of gut microbiota and the relative abundance of beneficial endophytes and decrease the relative abundance of harmful endophytes. The results indicated that the number of bacterial and fungal OTUs in the C0 group and C28 group was not different but increased in the DO28 group in contrast to the DO0 group (Supplement Figure 3(a)–3(b)). Meanwhile, the number of bacterial OTUs in the ASDO28 group increased compared with the ASDO0 in bacteria, but that of fungi decreased (Supplement Figure 3(b)). According to the Kruskal–Wallis rank-sum test, there was no significant difference in the relative abundance of *Lactobacillus*, *Ruminococcus*, *Alistipes*, *Aerococcus*, *Bacteroides*, *Lachnoclostridium*, *Anaerostipes*, *Parasutterella*, *Pyxidiophora*, *Cladosporium*, *Talaromyces*, *Rhodotorula*, *Filobasidium*, *Aspergillus*, *Mortierella*, *Penicillium*, *Cutaneotrichosporon*, and *Candida* in C0, ASDO0 and DO0 groups (*P* < 0.05); in addition, the relative abundance of *Bifidobacterium* of DO0 was significantly lower than that of C0 and ASDO0 groups (*P* > 0.05). However, DO28 increased the relative abundance of bacterial genera, including *Lactobacillus*, *Bifidobacterium*, *Ruminococcus*, and *Alistipes*, whereas decreased *Aerococcus*, *Bacteroides*, *Lachnoclostridium*, *Anaerostipes*, and *Parasutterella* compared with those in C28 and ASDO28 (Figure 3(a)). DO28 increased the relative abundance of fungal genera, including *Pyxidiophora*, *Cladosporium*, *Talaromyces*, *Rhodotorula*, *Filobasidium*, and decreased *Aspergillus*, *Mortierella*, *Penicillium*, *Cutaneotrichosporon*, *Candida*, compared with those in C28 and ASDO28 groups (Figure 3(b)). Our results also indicated that DO28 altered the gut microbiota structure in mice by increasing the beneficial bacteria *Lactobacillus murinus* [37], *Lactobacillus johnsonii* [38], *Bifidobacterium pseudolongum* [39], and *Lactobacillus reuteri* [40], and reducing harmful bacteria *Ochrobactrum anthropi* [41], *Aerococcus urinaeequi* [42], and *Clostridium* sp. cTPY-12 [43], compared with those in C28 and ASDO28 groups (Supplement Figure 4(a)).

**Figure 4:**
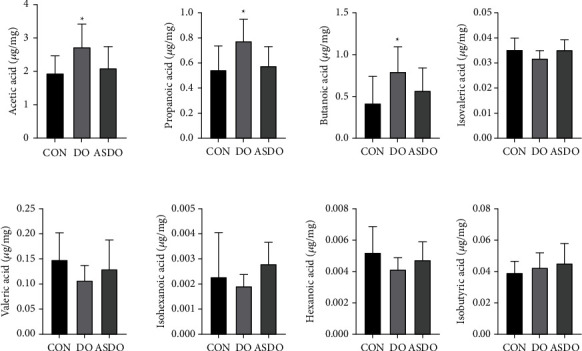
The concentration of eight SCFAs in fecal samples. ^*∗*^*P* ≤ 0.05.

SCFAs are one of the index components to evaluate gastrointestinal function [7]. We found that the *D. officinale* juice could effectively increase the content of SCFAs in mouse guts. Compared with the C28 and ASDO28 groups, the concentration of total SCFAs in the DO28 group was increased (Figure 4). Among them, acetic acid, propanoic acid, and butanoic acid contents of the DO28 group improved remarkably (*P* < 0.05), suggesting that DO28 may affect the intestinal environment to some extent by increasing SCFAs-producing bacteria, compared with those in C28 and ASDO28 groups. Taken together, *D. officinale* juice can effectively increase the contents of SCFAs.

These findings cumulatively suggest that *D. officinale* juice can effectively regulate gut microbiota by improving their diversity, increasing the relative abundance of beneficial endophytes and the contents of SCFAs, and reducing the relative abundance of harmful endophytes while these were not observed in sterilized (autoclaved) *D. officinale* juice.

### 3.3. *D. officinale* Endophytes May Colonize the Intestinal Tract of Mice and Modulate Gut Microbiota

**Figure 5:**
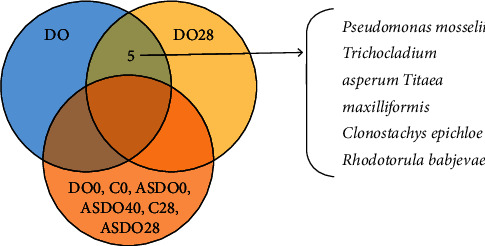
*D. officinale* endophytes may colonize in the intestinal tract of mice and modulate gut microbiota.

To examine which *D. officinale* endophytes may colonize in the intestinal tract of mice, we compared the microbiological community in the control mice and those administered with *D. officinale* juice and autoclaved *D. officinale* juice. We found three bacterial and 22 fungal genera in DO28 that were not found in DO0, C28, and ASDO28 groups. In addition, seven endophytic bacterial species were shared both in the DO and gut microbiota of normal mice. In addition, 24 kinds of endophytic fungal species were shared by DO and gut microbiota in normal mice. Among them, *Pseudomonas mosselii, Trichocladium asperum*, *Titaea maxilliformis*, *Clonostachys epichloe*, and *Rhodotorula babjevae* were found only in DO and DO28 groups while not in ASDO40, DO0, C0, C28, ASDO0, ASDO28 mice (Figure 5). Therefore, we hypothesize that the increase in beneficial gut microbiota after the administration of *D. officinale* fresh juice may be related to the *D. officinale* endophytes, including *Pseudomonas mosselii*, *Trichocladium asperum*, *Titaea maxilliformis*, *Clonostachys epichloe*, and *Rhodotorula babjevae*. These strains may colonize in the intestinal tract of mice and modulate gut microbiota.

## 4. Discussion

Autoclave steam sterilizing and ^60^Co-*γ* treatment could effectively reduce the OTU number and diversity of *D. officinale* endophytes and played a role in eliminating the interference of *D. officinale* endophytes. However, the results indicated that the relative abundance of *Ochrobactrum anthropi*, *Dokmaia monthadangi*, *Sporidiobolus pararoseus*, *Cladosporium delicatulum*, *Papiliotrema flavescens*, and *Rhodotorula babjevae* from CIDO25 increased, while that of *Ochrobactrum anthropi*, *Rhodococcus erythropolis*, *Lycoperdon utriforme*, *Cutaneotrichosporon cutaneum*, *Monascus pilosus*, and *Vishniacozyma* sp from ASDO40 increased. Previous studies have reported the radioresistance of *Ochrobactrum anthropi* and *Rhodotorula babjevae* [44, 45] and heat resistance of *Ochrobactrum anthropi* and *Rhodococcus erythropolis* [46, 47]. Therefore, we suggest that *Ochrobactrum anthropi* and *Rhodotorula babjevae* are potentially radiation-resistant while *Ochrobactrum anthropi* and *Rhodococcus erythropolis* are heat-resistant.

*D. officinale* can regulate gut microbiota and is closely related to the treatment of diseases [3]. Previous studies suggest that *D. officinale* can balance gut microbiota by improving the relative abundance of intestinal bacteria, such as *Ruminococcus*, *Clostridium*, and *Parabacteroides*, and decreasing the relative abundance of *Prevotella* and *Bacteroides* [11]. At the phylum level, Bacteroidota and Firmicutes were the predominant bacterial phyla, and Ascomycota was the predominant fungal phylum in all fecal samples. The predominant bacterial phyla were the same as those reported in a previous study [20]. *Lactobacillus* was the dominant bacterial genus in all the fecal samples, but the percent of relative abundance in DO28 was significantly higher than in ASDO28 and C28. Previous studies have reported the probiotic, exhibited immunomodulating [48], gastrointestinal protection [49], and antitumor [50] activities of *Lactobacillus*. The results of this study suggest that mice might produce more *Lactobacillus* to exert immunological, gastrointestinal protective, and antitumor effects after intragastric administration of DO. Interestingly, the relative abundance of *Lactobacillus johnsonii* from DO28 increased significantly compared with that in other samples. *Lactobacillus johnsonii* can promote T cell differentiation into T helper (Th)1/Th2/regulatory T (Treg) cells and play an important role in improving the balance between these cells [51]. Therefore, we suggest that *Lactobacillus johnsonii* might improve the bioaccessibility and bioavailability of functional components of *D. officinale* through the microbial-host metabolic pathway, thus maximizing its anti-immune function. However, future research is needed to determine whether these dominant gut microbial species can promote the utilization of effective components in *D. officinale*.

Gut microbes are closely related to SCFA utilization. SCFAs are key bacteria metabolites, in particularly acetic acid, propanoic acid and butanoic acid [4]. Interestingly, butanoic acid induces the differentiation of colonic Treg cells [52]. In our study, the SCFAs from the feces of mice were detected by GC-MS. As expected, DO-treated mice produced more butyrate (*P* < 0.05) and acetic acids (*P* > 0.05) than the control and ASDO groups. To know which are the primary butyrate-producing bacteria, the synthase that is responsible for butyrate synthesis should be investigated. This study indicated that DO cause an increase in the relative abundance of some SCFAs-producing bacteria *Lactobacillus johnsonii* [38], *Bifidobacterium pseudolongum* [39], and *Lactobacillus reuteri* [40], and decreased some pathobionts *Ochrobactrum anthropi* [41], *Aerococcus urinaeequi* [42], *Clostridium* sp. cTPY-12 [43]. Therefore, we suggest that the increase in the relative abundance of *Lactobacillus johnsonii*, *Bifidobacterium Pseudolongum*, and *Lactobacillus reuteri* might be closely related to the utilization of SCFAs by the DO endophytes. The increase in the relative abundance of fungi of *Rhodotorula babjevae* exhibited antimicrobial activity against different bacteria species [53], and *Lycoperdon utriforme* performed antioxidant activities [54]. However, there are no reports on SCFAs-producing fungal species, including *Rhodotorula Babjevae*, *Lycoperdon utriforme*, *Aspergillus Minisclerotigenes*, *Pyxidiophora arvernensis*, and other fungi.

Our results showed that the dominant bacterial and fungal genera of *D. officinale* were significantly different from gut microbiota in mice. However, *Fusarium* was a common dominant fungi genus identified both in *D. officinale* and fecal samples. Therefore, we provide evidence that a portion of gut microbiota in mice may be derived from *D. officinale* endophytes. Intriguingly, *Pseudomonas mosselii*, *Trichocladium asperum*, *Titaea maxilliformis*, *Clonostachys epichloe*, and *Rhodotorula babjevae* were found only in DO and DO28 and not in ASDO40, C0, DO0, C28, ASDO0, ASDO28 groups. Therefore, we speculated that these strains might colonize in the intestinal tract of mice and modulate gut microbiota after oral administration of *D. officinale* fresh juice for 28 days. These findings have important implications for understanding the increase in beneficial gut microbiota and metabolite SCFAs after the intake of *D. officinale* fresh juice, which may be attributed to the *D. officinale* endophytes. *Paenibacillus* sp. strain Aloe-11 exhibits intestine colonization ability and can improve forage fiber degradation, thus producing antibiotic activity against many pathogenic bacteria and fungi [17]. Zheng et al. (2020) reported that a portion of the intestinal microbes of the potato tuber moth might be derived from potato endophytic bacteria [18]. In addition, grass endophytic fungi could arrive at sheep guts and lower the fecal degradation rates [19]. However, the roles of *Pseudomonas mosselii*, *Trichocladium asperum*, *Titaea maxilliformis*, *Clonostachys epichloe*, and *Rhodotorula babjevae* were mainly reported as antifungal [55], plant disease resistance [56–58], while there have been only a few studies on the ability of *D. officinale* endophytes to colonize and transform host intestinal tract and a similar role in regulating gut microbiota in mice. According to this research, *D. officinale* juice could increase beneficial gut microbiota and metabolize SCFAs and may be related to *D. officinale* endophytes. Although high-throughput sequencing in this study has limitations, it can further study the colonization of plant endophytes in gut microbiota by macrogene and isolation and culture methods, but it provides a reference for this research idea. In addition, the red fluorescent protein can be applied to encode target strains through CRISPR/cas9 and instill gastric bacterial or fungal suspension into specific pathogen-free-induced pseudosterile mice, and then the colonization of the target strains can be verified by *in vivo* imaging [59–62].

## 5. Conclusions

This study showed that ASDO40 was more suitable for eliminating the interference of *D. officinale* endophytes. *D. officinale* juice could increase beneficial gut microbiota and metabolite SCFAs, which might be related to *D. officinale* endophytes. Additionally, the *D. officinale* endophytes, *Pseudomonas mosselii, Trichocladium asperum*, *Titaea maxilliformis*, *Clonostachys epichloe*, and *Rhodotorula babjevae* of might colonize in the intestinal tract of mice and modulate gut microbiota after oral administration with DO for 28 days. Whether these strains can colonize in the mouse intestine and participate in the regulation of gut microbiota in the treatment of diseases, need experimental verification, and our results provide a new approach for the treatment of diseases by colonizing and transforming plant endophytes into gut microbiota.

## Data Availability

The raw sequence data have been submitted to the NCBI Sequence Read Archive (SRA) under accession nos. PRJNA819231 and PRJNA819223.

## Supplementary Materials

Supplementary Materials Figure S1: distribution of (A) bacteria and (B) fungi in phylum, class, order, family, genus, and species in *D. officinale* samples. Figure S2: alpha indices of bacteria samples at the operational taxonomic unit level. (A) Shannon; (B) Chao; alpha indices of fungi samples; (C) Shannon; (D) Chao (*P* < 0.05^*∗*^; *P* ≤ 0.01^*∗∗*^; *P* ≤ 0.001^*∗∗∗*^). Figure S3: Venn diagram analysis for unique and shared operational taxonomic units among (A) bacteria and (B) fungi in *D. officinale* and fecal samples of experimental mice. Figure S4: community heatmap analysis of bacteria (A) and fungi (B) at the species levels.
