# Supplementary material for: Dendrobium officinale Endophytes May Colonize the Intestinal Tract and Regulate Gut Microbiota in Mice
Source: Evid Based Complement Alternat Med. 2022 Aug 11;2022:2607506. doi: 10.1155/2022/2607506 (PMC9388241; doi:10.1155/2022/2607506)
Supplement: Supplementary Materials — Figure S1: distribution of (A) bacteria and (B) fungi in phylum, class, order, family, genus, and species in D. officinale samples. Figure S2: alpha indices of bacteria samples at the operational taxonomic unit level. (A) Shannon; (B) Chao; alpha indices of fungi samples; (C) Shannon; (D) Chao (P < 0.05∗; P ≤ 0.01∗∗; P ≤ 0.001∗∗∗). Figure S3: Venn diagram analysis for unique and shared operational taxonomic units among (A) bacteria and (B) fungi in D. officinale and fecal samples of experimental mice. Figure S4: community heatmap analysis of bacteria (A) and fungi (B) at the species levels. [file 2607506.f1.pdf]

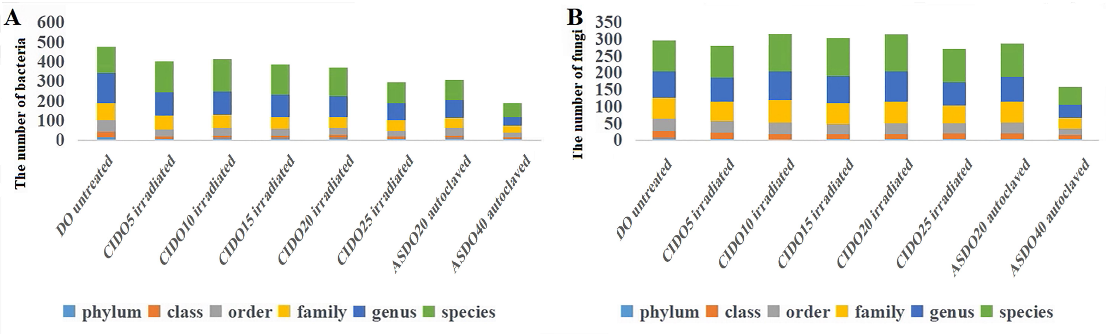

Figure S1: Distribution of (A) bacteria and (B) fungi of in phylum, class, order, family, genus, and species in *D. officinale* endophyte juice samples.

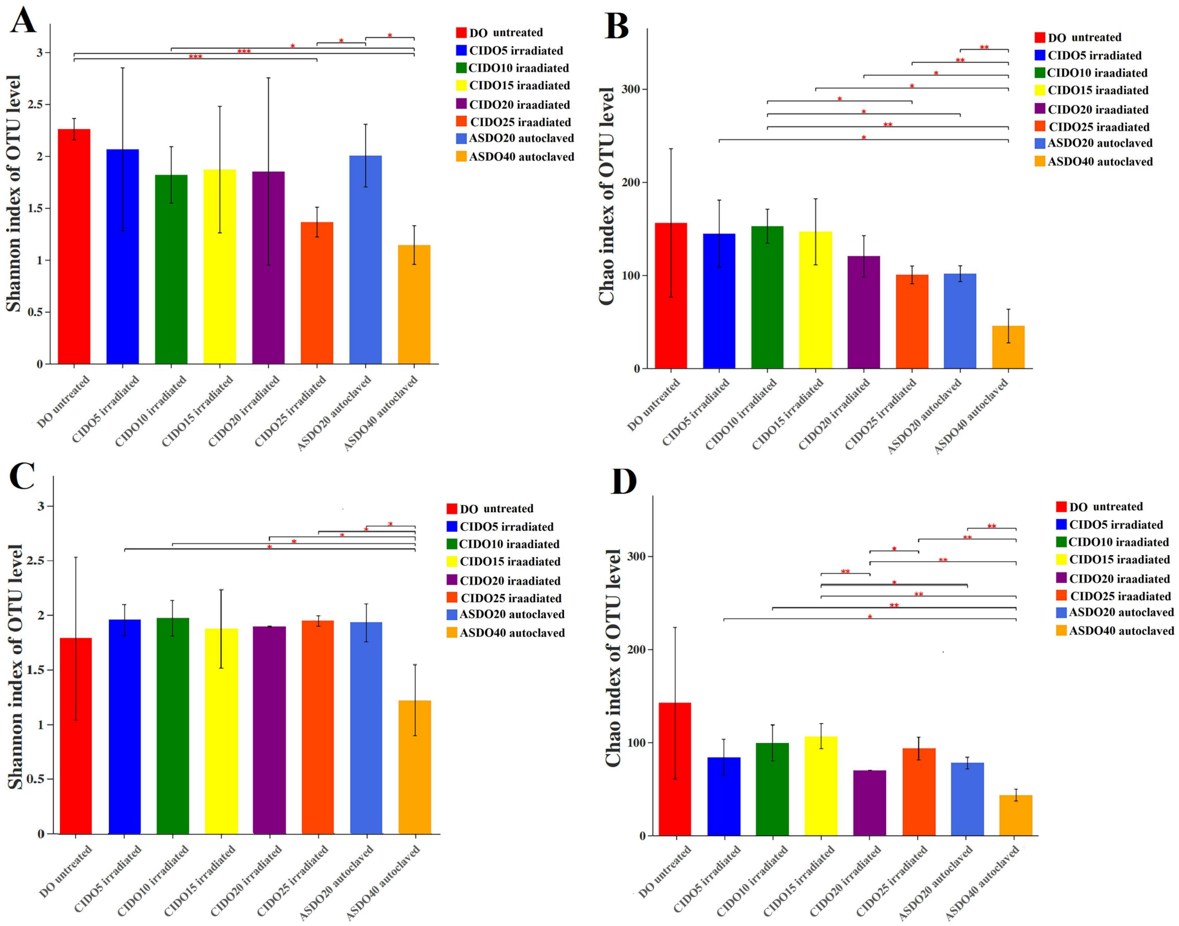

Figure S2: Alpha indices of bacteria samples at operational taxonomic unit (OTU) level. (A) Shannon, (B) Chao; alpha indices of fungi samples, (C) Shannon, (D) Chao (P≤0.05 *; P≤0.01 **; P≤0.001 ***).

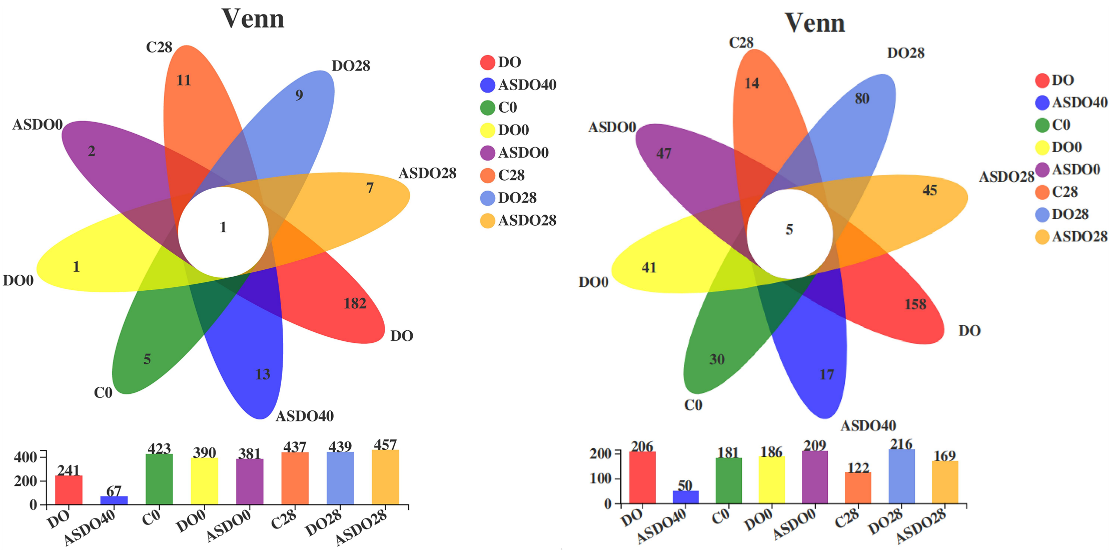

Figure S3: Venn diagram analysis for unique and shared operational taxonomic units among (A) bacteria and (B) fungi in *D. officinale* and fecal samples of experimental mice.

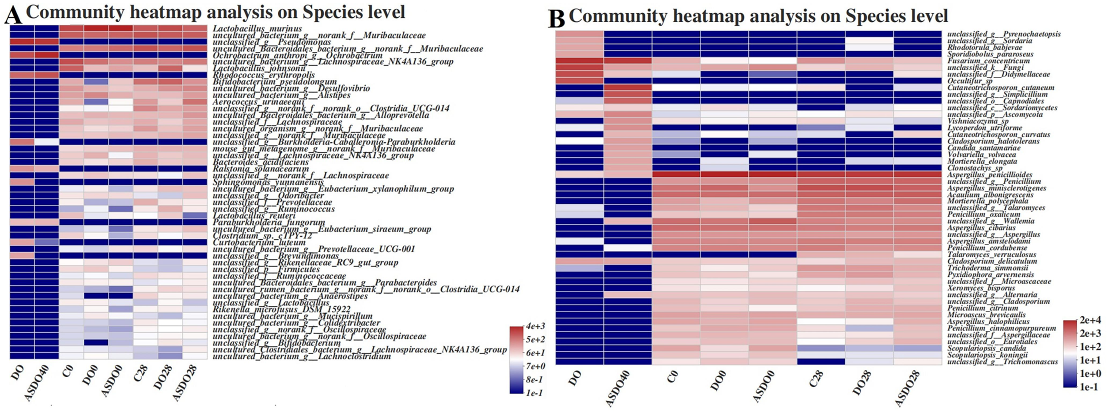

Figure S4: Community heatmap analysis of bacteria (A) and fungi (B) at the species levels.
